# Analysis of Persian Bioinformatics Research with Topic Modeling

**DOI:** 10.1155/2023/3728131

**Published:** 2023-04-17

**Authors:** Fezzeh Ebrahimi, Mohammad Dehghani, Fatemah Makkizadeh

**Affiliations:** ^1^Department of Scientometrics, Faculty of Social Sciences, Yazd University, Yazd, Iran; ^2^School of Electrical and Computer Engineering, University of Tehran, Tehran, Iran; ^3^Faculty of Social Sciences, Yazd University, Yazd, Iran

## Abstract

**Purpose:**

As a scientific field, bioinformatics has drawn remarkable attention from various fields, such as information technology, mathematics, and modern biological sciences, in recent years. The topic models originating from the field of natural language processing have become the focus of attention with the rapid accumulation of biological datasets. Thus, this research is aimed at modeling the topic content of the bioinformatics literature presented by Iranian researchers in the Scopus Citation Database. *Methodology*. This research was a descriptive-exploratory study, and the studied population included 3899 papers indexed in the Scopus database, which had been indexed in this database until March 9, 2022. The topic modeling was then performed on the abstracts and titles of the papers. A combination of LDA and TF-IDF was utilized for topic modeling. *Findings*. The data analysis with topic modeling resulted in identifying seven main topics “Molecular Modeling,” “Gene Expression,” “Biomarker,” “Coronavirus,” “Immunoinformatics,” “Cancer Bioinformatics,” and “Systems Biology.” Moreover, “Systems Biology” and “Coronavirus” had the largest and smallest clusters, respectively.

**Conclusion:**

The present investigation demonstrated an acceptable performance for the LDA algorithm in classifying the topics included in this field. The extracted topic clusters indicated excellent consistency and topic connection with each other.

## 1. Introduction and Problem Statement

Bioinformatics emerged in the early 2000s in response to the exponentially increasing data generated by whole genome and protein sequencing techniques. In molecular biology, pioneering work on mapping and comparing protein and gene sequences led to an intensive phase that can be referred to as database mining and the development of computer algorithms that are efficient and reliable. Nowadays, bioinformatics has reached a high level of maturity and sophistication as a science. The field of bioinformatics is now being used in structural biology, computational chemistry, genetics, molecular biology, the pharmaceutical industry, and pharmacology.

Information technology, computer science, mathematics, statistics, and biotechnology are incorporated into bioinformatics to provide methods to extract information and discover biological processes [1]. The term “Bioinformatics” was used for the first time in 1968 and defined in 1978. Moreover, bioinformatics is recognized as “Computational Biology.” However, speaking more precisely, computational biology largely deals with the modeling of biological systems [2]. The era of new biology emerged with the generation of some sciences, such as bioinformatics and computational biology [3]. In fact, bioinformatics appears as the application of computational tools and the analysis and interpretation of biological data. Therefore, it seems essential to manage the data of biology and modern medical sciences [4]. The principal components of bioinformatics are as follows:

1. Development of software tools and algorithms
2. Biological data analysis and interpretation using various software tools and specific algorithms [2].

Considering the importance of bioinformatics, research in this field has attracted the attention of researchers. Due to the importance of issues and topics related to the field of bioinformatics as an interdisciplinary science as well as to obtain the structure, development process, and changes made in this field, the use of an efficient method seems necessary to provide a potential to discover the latent topics in the heart of the studies through the latent relationships between the topics.

Topic modeling is a statistical model to discover latent “topics,” which happens in a set of documents through machine learning. The core significance of topic modeling is to discover the patterns of words and the quality of documents connected with similar patterns [5]. Topic modeling techniques act beyond a classification or clustering approach and have the potential to model a biological object based on latent “topics” that can reflect the underlying biological meaning more exclusively [6]. Furthermore, the topic modeling technique may be applied to reveal the clustering of the concepts of scientific fields, classify documents based on subjects, and discover prominent patterns and emerging events. This type of knowledge identification highly matters in determining research priorities, developing policy makers' strategic plans, getting knowledge of the gap in Iranian research topics, and the interdisciplinary effects of this field and provides the scientific-research planners and policymakers with a paved path to develop strategic plans. In the present study, the LDA algorithm was used to determine the development and trend of the topic field of bioinformatics based on the documents published by Iranian researchers in the Scopus database, and it was designed to identify the most important subject areas, the most important topics based on the TF-IDF weight, and the topic clusters of publications in the field of bioinformatics based on the topic modeling algorithm. Several studies have been published that used various scientometrics approaches in biomedicine. Bansard et al. analyzed the literature in bioinformatics and medical informatics to identify future common trends between the two research areas [7]. Moreover, Xu et al. performed a study by utilizing scientometric tools to identify hot topics and draw the bioinformatics and neuroscience data map [8]. In addition, Heo et al. reviewed and examined 46 bioinformatics journals in a five-year period from 1996 to 2015 using the author-conference-topic (ACT) model [9]. Sudhier and Roselin studied the bioinformatics research in India to evaluate the growth of research in this field by examining top-rated journals and authors, journals' citations, h-index, and p-index and drawing their maps during 2011-2019 [10]. Furthermore, Kiani et al. conducted a study to draw a knowledge map of concepts of bioinformatics based on the papers presented in the InCites Clarivate database [11]. This research was performed using a descriptive-analytical method through coword analysis techniques. Moreover, Gupta et al., in a study entitled “Prediction of the research process using the LDA-based topic modeling,” focused on discovering paradigms as predictors with the relevance of each topic through topic modeling techniques such as LDA with the BoW [12]. Many previous studies have evaluated bioinformatics with scientometric algorithms and word analysis, and some researchers have discovered latent patterns using text mining algorithms [10–12]. Moreover, some other researchers focused on the topic modeling of scientific productions in databases. However, no research deals with text mining and the discovery of Iranian research's bioinformatics topics using the topic modeling method with the LDA algorithm.

## 2. Methodology

This research was a descriptive-exploratory study. The studied population included 3899 papers indexed in the Scopus database, which had been indexed in this database until March 9, 2022. The following search strategy was used with limiting organizational affiliation to Iran in the keywords, abstract, and title fields. It should be noted that the same search strategy has been used in a study conducted by Armenta et al. [13].

(TITLE-ABS-KEY (bioinformatics) OR TITLE-ABS-KEY ((computational AND genomics)) OR TITLE-ABS-KEY (“computational biology”) OR TITLE-ABS-KEY (computational AND transcriptomics)) OR TITLE-ABS-KEY ((computational AND proteomics)) OR TITLE-ABS-KEY ((computational AND protein)) OR TITLE-ABS-KEY ((computational AND DNA)) OR TITLE-ABS-KEY ((computational AND RNA)) OR TITLE-ABS-KEY (“Systems biology”) AND AFFIL (Iran))

This research was performed in three steps, as explained in the following:

(1) Preprocessing
The documents needed to be preprocessed to review a large set of texts to store the information in an appropriate data structure for further processing. This stage involved the following steps for preprocessing of abstracts and titles of papers.
  - *Removal of Empty Records*. Abstract and title records as NULL were removed at this stage
  - *Punctuation Removal.* In this step, punctuation marks were removed since some word-embedding models do not support them
  - *Tokenization*. This is a method to break texts into smaller units, known as tokens
  - *Stop Word Removal*. These words do not add much value to the meaning of the content. Therefore, they were removed in the preprocessing stage
  - *Lemmatization*. This step reduces conjunction words and contributes to retrieving or fetching valid and essential words [12]. At this stage, the words were converted to their roots
(2) Conversion of texts to numeric vectors
The next step is vectorization because most machine learning techniques only work with numerical data, so in order to use and apply these techniques to textual data, it is necessary to convert the texts into a set of numbers. In this step, the TF-IDF converts the raw textual data into a matrix-based data set.
(3) Topic modeling
Topic modeling is an unsupervised machine learning strategy with the potential to examine a set of documents, identify words, and discover patterns within them; thereby, it can cluster words and comparative phrases. Accordingly, it can distinguish a set of sets in an appropriate manner. In summary, topic modeling algorithms produce a set of expressions and words that are believed to be relevant, allowing the reader to comprehend the relationships while classifying the topics.

The input from the TF IDF in the previous step was given to the LDA algorithm in this step so it can model the topics. LDA is a probabilistic approach to topic modeling. In other words, it is a hierarchical Bayesian probability generation model for discrete data collection that relies on the assumption that the document's words and topics are consistent. The subsequent topics are displayed as a discrete distribution of the entire phrase in the document, and it models the body as a discrete distribution of the entire subject. Since the equilibrium is determined at the document level, this model is regarded as being superior to others that also use competitive dispersion. Gensim, a Python library used for topic modeling, document indexing, and similarity retrieval with large corpora, was used in this investigation to implement LDA.

It should be noted that the Pandas, Gensim, Numpy, NLTK, and Sklearn packages were employed to code this research project in Python using the Colab environment.

## 3. Results

A total of 3899 papers were retrieved based on the search made in the Scopus database. The NULL data were removed from the dataset. The title fields were not null in any of the records, and 24 abstract fields were null, which were deleted; thus, 3875 records were investigated for analysis.

### 3.1. Most Important Applied Words Based on the Frequency of Scientific Productions in the Field of Bioinformatics

**Figure 1:**
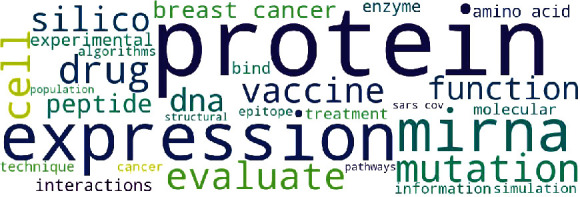
Cloud of words with the highest frequencies.

According to the results obtained from the analysis of bioinformatics papers with the organizational affiliation limited to Iran in the Scopus database, the words “protein,” “expression,” “mirna,” “mutation,” and “drug” were identified as the five words with higher frequencies.  Figure 1 indicates the cloud of words with the highest frequencies.

### 3.2. Most Important Applied Words Based on the Weighted Frequency

The data in Table 1 contains the ten most important words based on the TF-IDF weight. According to the data analysis, the “mir,” “expression,” and “cancer,”, respectively, with weights of 105.24, 85.47, and 83.80, had the highest weights in the scientific productions of bioinformatics.

**Figure 2:**
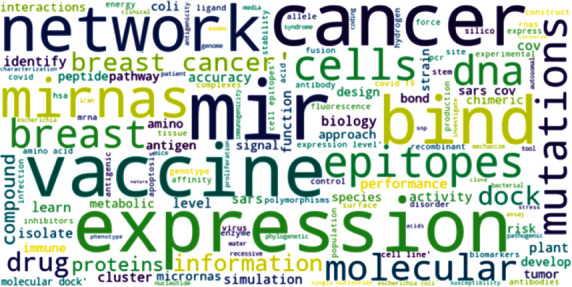
Cloud of top 100 words based on the TF-IDF weights.

Moreover, Figure 2 indicates the cloud of the top 100 words based on the TF-IDF weights.

### 3.3. Percentage of Scientific Productions in the Field of Bioinformatics Based on Seven Topic Clusters in the Scopus Database

**Figure 3:**
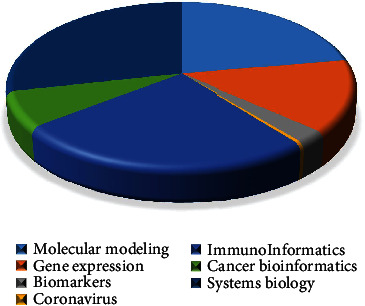
Frequency of the topics of scientific productions in the field of bioinformatics.

Figure 3 demonstrates the percentage of bioinformatics productions based on classifying seven topic clusters in the Scopus database with the organizational affiliation limited to Iran. Accordingly, the topics “systems biology,” “immunoinformatics,” and “molecular modeling” have the highest percentages of scientific productions, with approximately 28%, 25%, and 22%, respectively. In contrast, the topic “coronavirus” has the lowest percentage.

### 3.4. Topic Clusters of Bioinformatics Productions with the Organizational Affiliation Limited to Iran Based on the Topic Modeling Algorithm

The data in Table 2 indicates the implementation of the topic modeling algorithm on the scientific productions in the field of bioinformatics with the organizational affiliation limited to Iran based on the seven topic clusters.

**Figure 4:**
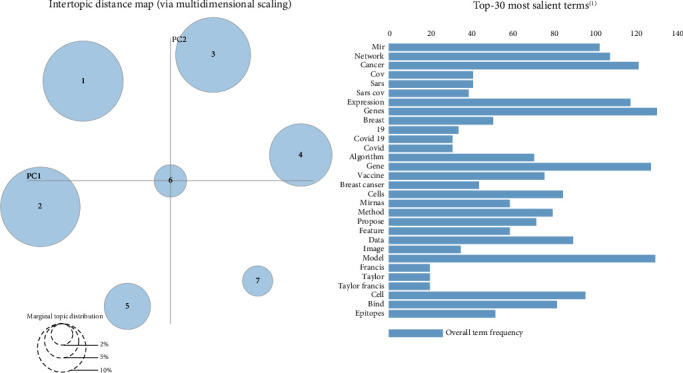
Topics formed of the scientific productions in the field of bioinformatics with the organizational affiliation limited to Iran.

Moreover, according to Figure 4, the seven topic clusters have no commonality or overlap. Therefore, it can be said that the topics are well separated by this algorithm.

**Figure 5:**
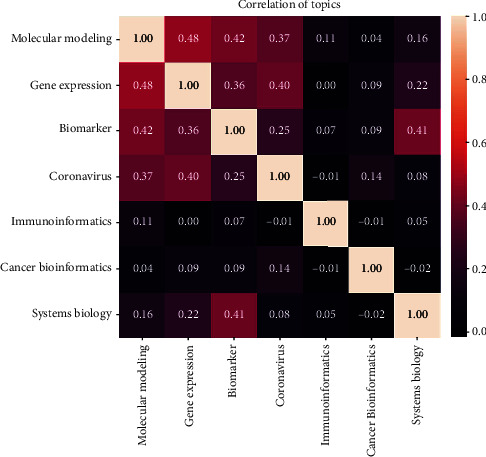
Correlation of topics.

Afterward, the author's keywords were used as features, and their correlation in each topic was determined using the BOW method. As mentioned before (Figure 3), in the topic modeling method with the LDA algorithm, each article is placed within the most relevant topic. Considering that each article has about 5-7 keywords, this section examines the correlation of the top 1000 keywords with other topics (Figure 5). The findings indicated that the topics “gene expression” and “molecular modeling” have the highest correlation, with a value of 0.48. These are followed by “biomarker” and “molecular modeling,” with correlation values of 0.42 and 0.41, respectively.

**Figure 6:**
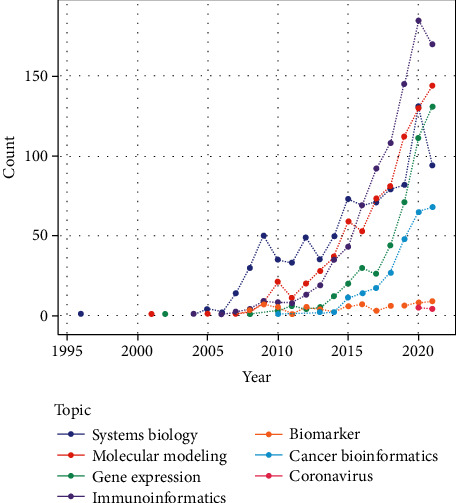
Publishing trend of topics of articles in the field of bioinformatics.

The next stage involved obtaining Iranian researchers' papers published in the field of bioinformatics. Most papers in this field were published in 2020, as shown in Figure 6. According to this figure, the topic “immunoinformatics” has the highest rate of publications. After this topic, “molecular modeling” has had the highest publication rate.

**Figure 7:**
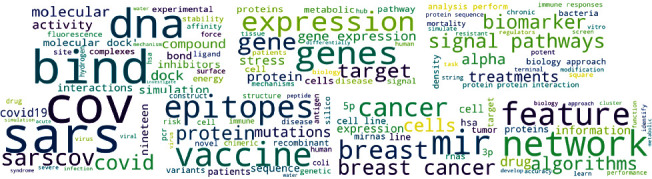
Topics identified by combining LDA+TF_IDF algorithms.

Figure 7 indicates the top word clouds in the seven mentioned topics, which are explained in the following.

#### 3.4.1. Topic 1: Molecular Modeling

All theoretical and computational methods used to model or mimic the behavior of molecules are considered molecular modeling [14]. Molecular modeling methods are now used routinely to investigate the structure, dynamics, surface properties, and thermodynamics of inorganic, biological, and polymeric systems [15]. The methods are used in the fields of computational chemistry, drug design, computational biology, and materials science to study molecular systems ranging from small chemical systems to large biological molecules and material assemblies. The most straightforward calculations can be performed manually, but, inevitably, computers are required to perform molecular modeling of any reasonably sized system.

#### 3.4.2. Topic 2: Gene Expression

A wide range of important biological processes, including tissue homeostasis, immunity, cellular stress responses, organism growth, and cell differentiation, depend critically on the control of gene expression. In the past few years, significant progress has been made in our understanding of the molecular mechanisms underlying transcription, the function of chromatin and its modifications, the role of enhancers, and the role of noncoding RNAs [16]. The process of using a gene's information to produce a functional product is known as gene expression. In conjunction with nonamino acid products like rRNA, tRNA, sRNA, and mRNA, amino acids are the primary products of genes. All eukaryotes and prokaryotes (bacteria, etc.) perform gene expression.

#### 3.4.3. Topic 3: Biomarkers

Applications for biomarkers may include early disease detection, tracking the course of the disease, and selecting the most effective course of therapy. Disease-related and drug-related biomarkers are the broad categories into which biomarkers can be broadly divided. The need to identify the unique characteristics of the patient is growing as personalized treatment strategies for patients become popular. These details enable medical professionals to appropriately schedule their patients' care and administrate accurate care [17].

#### 3.4.4. Topic 4: Coronavirus

By causing the COVID-19 illness, the newly discovered pathogen SARS-CoV-2 has sparked a global pandemic crisis. The unique and newly-seen genetic makeup of SARS-CoV-2 has made biological research face some obstacles. However, utilizing bioinformatics tools and techniques, researchers have successfully interpreted this viral genomic architecture. Such interpretations appear essential for ongoing research on the COVID-19 pandemic and the introduction of a SARS-CoV-2 vaccine that could protect public health [18].

#### 3.4.5. Topic 5: Immunoinformatics

Immunoinformatics, otherwise known as computational immunology, is the interface between computer science and experimental immunology. This concept represents the use of computational methods and resources for understanding immunological information. Immunoinformatics not only helps deal with vast amounts of data but also plays a significant role in defining new hypotheses related to immune responses [19]. Immunogenomics, immune-proteomics, epitope prediction, and in silico vaccination are different areas of computational immunological research. Recently, systems biology approaches have been applied to investigate the properties of the dynamic behavior of an immune system network [20].

#### 3.4.6. Topic 6: Cancer Bioinformatics

Cancer bioinformatics is a critical part of the systems clinical medicine in cancer and the core tool and approach to carry out cancer investigations in systems clinical medicine. The approach to studying the genetic basis of cancer is undergoing a revolution. Rather than focusing on individual genes, scientists are now exploring substantial components of the expressed genome. The wealth of molecular information being generated from the laboratory as well as the volume of data being stored in the patient record is continuing to increase at an astounding rate. Finding new ways to integrate these data has been crucial to developing novel insights into cancer genetics. Therefore, bioinformatics, the convergence of biology, information science, and computation, is continuing to emerge as a crucial component of cancer biology research [21].

#### 3.4.7. Topic 7: Systems Biology

With 1110 records, the largest cluster relates to the field of systems biology. Model, algorithm, proposal, method, data, network, feature, information, classification, etc. are among the terms with significant weight in this cluster. Due to the enormous amount of data obtained through biotechnology, the computational and mathematical analysis and modeling of biological systems are highly challenging. Research on intricate interactions between biological systems is performed in this interdisciplinary area of biology-based studies. In contrast to the conventional reductionist approach, it takes a holistic approach to biological research [22].

## 4. Discussion

The main objectives of this study included topic modeling methods based on finding latent topics, topic-based document interpretation, and ultimately organizing and categorizing the texts under study. With these objectives in mind, the current study used the topic modeling method to evaluate the scientific outputs of Iranian researchers in the field of bioinformatics using the LDA and TF IDF algorithms.

The findings of the study showed that the five terms “protein,” “gene,” “method,” “model,” and “application” were used the most frequently in the bioinformatics studies carried out by Iranian researchers. In terms of TF-IDF weights, the words “MIR,” “model,” “gene,” “vaccine,” and “binding” had the highest weights in the body of the papers. As can be seen, the words “gene” and “model” are among the most frequent and heavily weighted words in both cases, indicating the importance of these words in the field of bioinformatics. Some definitions claim that informatics emerged as a result of the enormous amount of genomic data that has been generated and the need to properly store, retrieve, and analyze it. Gene discovery, genome assembly, etc. may be cited as some of the most notable research efforts in the field of bioinformatics. The steps in the bioinformatics method known as building a computational model and solving a computational modeling problem after data collection are known as essential steps.

MicroRNA and gene expression were among the subjects with the highest frequency rates, according to research [11].

In addition, the LDA topic modeling algorithm was used in this paper to identify seven topics, including coronavirus, gene expression, systems biology, molecular modeling, cancer bioinformatics, and immunoinformatics.

The fields of biology, applied statistics and mathematics, computer science, and chemistry are all clearly used in the field of bioinformatics, as can be seen in the clusters. In other words, bioinformatics is used to analyze biological issues using statistical and mathematical methods within a computer.

In terms of the share of productions devoted to each topic, systems biology and immunoinformatics have experienced the highest frequency rates, whereas coronavirus has experienced the lowest frequency rates. The study area of bioinformatics focuses on the computational analysis of biological data. Systems biology is a field of study that focuses on comprehending entire biological systems like protein complexes, metabolic pathways, or gene regulatory networks, as opposed to bioinformatics, which focuses on single genes or proteins. As a result, it is referred to as a branch of bioinformatics. According to literature [9], the systems biology cluster supports the idea that the role of the auxiliary fields of bioinformatics, such as conceptual, mathematical, and systematic biology, is progressively growing. This cluster also has the most papers in it, which is in line with his findings.

The field of immunoinformatics accounted for the highest proportion of productions and the topic's highest growth trend. Computational immunology, also known as immunoinformatics, has recently emerged as a significant and cutting-edge field in the sciences of analysis, modeling, immune system function prediction, vaccine design, allergenicity research, and pharmaceutical discoveries. Due to its interaction with the human genome project and other organisms, this science has not only aided in the acceleration of scientific research but also resulted in the achievement of a great deal of information pertaining to immunology. In actuality, immunoinformatics serves as a link between computational methods and experimental experiments. Because of this field's direct connection to global health and the fact that it speeds up and lowers the cost of scientific research from a different angle, its successes are of high strategic importance.

Since the coronavirus outbreak began in 2019, papers in this area have also started to be written as of that time. Therefore, it is not surprising that there are fewer papers on this topic than on others.

The COVID-19 pandemic's emergence, rapid geographic spread (to more than 200 countries), and the WHO's designation of the outbreak as a public health emergency of international concern have prompted researchers to take advantage of bioinformatics tools and techniques in order to quickly develop new diagnostics and vaccines. As a result, the coronavirus appears to form a distinct cluster in the field of bioinformatics due to the significance of this issue.

## 5. Conclusion

This study identified the bioinformatics topic clusters among Iranian researchers using text-mining techniques and topic modeling tools. These subjects include systems biology, immunoinformatics, cancer bioinformatics, biomarkers, coronavirus, gene expression, and molecular modeling. The biggest and smallest clusters belonged to the topics of systems biology and coronavirus, respectively. The clustering of texts requires the extraction and preservation of semantic information, and one of the characteristics of this extraction is the use of features that have this attribute. As a result, the latent Dirichlet allocation (LDA) model was employed in this paper as a semantic analysis method for feature extraction. The classification of topics in this field has shown a passable performance for the LDA algorithm. The extracted topic classes are well contained and have strong subject connections. Despite the fact that the words in each topic heading may not be identical, they are undoubtedly related. A topic model, in contrast to conventional clustering, enables one to obtain data from multiple clusters rather than just one. These characteristics might be advantageous in the field of bioinformatics.

In general, the findings of this research may, on the one hand, provide a better context for developing research programs and policy, and, on the other hand, they may lead to further recognition and use of topics that have been given more thought.

The intellectual structure of the bioinformatics field in Iran can be inferred from the outputs indexed in the global database and used to predict future research topics and trends by identifying the outstanding topics, which in some way indicate the main trends of the research in a subject area. The gaps in the literature can also be found. In addition, the modeling of topics of bioinformatics articles worldwide and comparing another algorithm with that of the present study is suggested for future research.

## Figures and Tables

**Table 1:** Frequency of the ten most important words in published articles in the field of bioinformatics based on TF-IDF weight.

No.	Word	Weight
1	mir	105.24
2	Expression	85.47
3	Cancer	83.80
4	Vaccine	82.22
5	Bind	67.16
6	Network	61.68
7	mirnas	61.34
8	Epitopes	57.19
9	Cells	55.32
10	Feature	53.56

**Table 2:** Seven topic categories of scientific productions in the field of bioinformatics.

No.	Topics	Words
1	Molecular modeling	Bind, DNA, molecular, protein, structure, dock, activity, amino, interactions
2	Gene expression	Genes, expression, gene expression, mirnas, pathways, cancer, mirna
3	Biomarkers	Alpha, biomarkers, biological approach, protein-protein interaction, protein sequence, simulation, bacteria, pathways signal, biomarker, treatments
4	Coronavirus	Cov, sars, sars cov, covid, covid 19, coronavirus, drug, virus, viral
5	Immunoinformatics	Vaccine, epitopes, sequence, mutations, patients, epitope, mutation, and recombinant
6	Cancer bioinformatics	mir, cancer, breast, breast cancer, cells, tumor, 5p, target, 3p, cell line, tissue, and tumors
7	Systems biology	Model, algorithm, method, data, base, algorithms, network, feature, approach, biological, prediction, methods, computational, process, systems, and optimization

## Data Availability

The data can be downloaded from the Scopus database.

## Abbreviations

LDA: Latent Dirichlet allocation
TF: Term frequency
IDF: Inverse document frequency
BoW: Bag of words
ACT: Author-conference-topic.
